# Netupitant, a Potent and Highly Selective NK_1_ Receptor Antagonist, Alleviates Acetic Acid-Induced Bladder Overactivity in Anesthetized Guinea-Pigs

**DOI:** 10.3389/fphar.2016.00234

**Published:** 2016-08-04

**Authors:** Stefano Palea, Véronique Guilloteau, Moéz Rekik, Emanuela Lovati, Marc Guerard, Maria-Alba Guardia, Philippe Lluel, Claudio Pietra, Mitsuharu Yoshiyama

**Affiliations:** ^1^UROsphereToulouse, France; ^2^Palea Pharma and Biotech ConsultingToulouse, France; ^3^Research and Preclinical Development, Helsinn Healthcare S.A.Lugano, Switzerland; ^4^Department of Urology, University of Yamanashi Graduate School of Medical ScienceChuo, Japan

**Keywords:** afferent, cystometry, detrusor muscle, intersticial cystitis, L-733,060, substance P, tachykinins

## Abstract

**Introduction.** Tachykinins potently contract the isolated urinary bladder from a number of animal species and play an important role in the regulation of the micturition reflex. On the guinea-pig isolated urinary bladder we examined the effects of a new potent and selective NK_1_ receptor antagonist (netupitant) on the contractions induced by a selective NK_1_ receptor agonist, SP-methylester (SP-OMe). Moreover, the effects of netupitant and another selective NK_1_ antagonist (L-733,060) were studied in anesthetized guinea-pigs using two experimental models, the isovolumetric bladder contractions and a model of bladder overactivity induced by intravesical administration of acetic acid (AA).

**Methods and Results.** Detrusor muscle strips were mounted in 5 mL organ baths and isometric contractions to cumulative concentrations of SP-OME were recorded before and after incubation with increasing concentrations of netupitant. In anesthetized female guinea-pigs, reflex bladder activity was examined under isovolumetric conditions with the bladder distended with saline or during cystometry using intravesical infusion of AA. After a 30 min stabilization period, netupitant (0.1–3 mg/kg, i.v.) or L-733,060 (3–10 mg/kg, i.v.) were administered. In the detrusor muscle, netupitant produced a concentration-dependent inhibition (mean pK_B_ = 9.24) of the responses to SP-OMe. Under isovolumetric conditions, netupitant or L-733,060 reduced bladder contraction frequency in a dose-dependent manner, but neither drug changed bladder contraction amplitude. In the AA model, netupitant dose-dependently increased intercontraction interval (ICI) but had no effect on the amplitude of micturition (AM). L-733,060 dose-dependently increased ICI also but this effect was paralleled by a significant reduction of AM.

**Conclusion.** Netupitant decreases the frequency of reflex bladder contractions without altering their amplitude, suggesting that this drug targets the afferent limb of the micturition reflex circuit and therefore may be useful clinically in treating bladder overactivity symptoms.

## Introduction

The neuropeptide Substance P (SP) is widely distributed in the body of mammals and displays a wide variety of physiological functions, acting mainly through stimulation of NK_1_ receptors. NK_1_ receptor antagonists have great potential in the treatment of a variety of pathologies such as pain, depression, asthma and emesis. Three NK_1_ receptor antagonists are on the market today as antiemetics, i.e., aprepitant (Curran and Robinson, 2009), rolapitant (Schwartzberg et al., 2015) and netupitant in combination with the 5-HT_3_ receptor antagonist palonosetron (Lorusso et al., 2015). SP is a major neuropeptide in capsaicin-sensitive primary afferent neurons innervating the mammalian urinary bladder (Sharkey et al., 1983). An increasing number of pharmacological studies using selective tachykinin receptor antagonists have demonstrated that these peptides play an important role in the sensory control of bladder contractions. The majority of studies on the role of NK_1_ receptors in the urinary bladder function have been performed in rats. It has been reported that intrathecal administration of NK_1_ receptor antagonists increases bladder capacity in anesthetized rats, whereas NK_2_ antagonists are ineffective (Lecci et al., 1993). In other study (Zhang et al., 2008), an intrathecal injection of L-733,060, a selective and potent NK_1_ antagonist, was ineffective in normal rats, but blocked the spinal micturition reflex in the majority of rats with chronically transected spinal cord. More importantly, however, despite the fact that the pharmacology of NK_1_ receptors in guinea-pigs is very similar to that in humans, few studies have been conducted to examine the effects of an NK_1_ receptor antagonist on the urinary bladder function in this animal species. It was previously shown that spinal NK_1_ receptors are involved in the micturition reflex in spinalized, anesthetized guinea-pigs (Palea and Pietra, 1999). The selective NK_1_ receptor antagonist, TAK-637, increased the volume threshold without affecting voiding pressure in anesthetized male guinea-pigs (Doi et al., 1999) and it was suggested that these effects were mediated, at least in part, by an action on the spinal cord (Kamo et al., 2000). These studies, besides indicating that cystometry in guinea-pigs is a good model to study the function of NK_1_ receptors on the bladder function, showed that the mechanism of action of NK_1_ antagonists clearly differs from that of antimuscarinics (Kamo et al., 2000). Interestingly, it was previously reported that the selective NK_1_ receptor antagonists aprepitant (Green et al., 2006) and serlopitant (Frenkl et al., 2010) were slightly effective in the treatment of OAB in female patients. This study, therefore, was conducted to evaluate the effects of netupitant *in vitro* and *in vivo* on the urinary bladder function. Extensive pharmacological characterization of netupitant was previously reported (Rizzi et al., 2012). Since functional NK_1_ receptors were demonstrated in guinea-pig urinary bladder smooth muscle (Longmore and Hill, 1992), the first aim of the present study was to evaluate the effects of netupitant on guinea-pig isolated detrusor muscle contracted with the selective NK_1_ receptor agonist, substance P methylester (SP-OMe). The functional selectivity of netupitant on the urinary bladder was tested by investigating its effects on contractions induced by the muscarinic agonist, carbachol. The second aim was to compare the effects of netupitant and L-733,060, another potent NK_1_ receptor antagonist (Rupniak et al., 1996; Seabrook et al., 1996), in two experimental models for examining *in vivo* bladder function in anesthetized guinea-pigs. The first paradigm, the isovolumetric model, was useful for a first screening of the doses of NK_1_ antagonists having a clear effect on the bladder activity. The second paradigm, an animal with bladder overactivity induced by intravesical infusion of diluted (0.2%) acetic acid (AA), is generally considered as an adequate model of irritative bladder hyperreflexia.

## Materials and Methods

### General

All experiments were carried out at UROsphere, Toulouse, France, in strict accordance with the recommendations from the European Community Council Directive 86/609/EEC. UROsphere is titular of a license from the French Ministry for Agriculture and Fisheries which allows using the type of animal models as described below.

Adult female Dunkin-Hartley guinea-pigs were obtained from Charles River Laboratories (L’Arbresle, France). Animals were delivered to the laboratory at least 5 days before the experiments during which time they acclimatized to the animal house conditions. Guinea-pigs were housed in groups of 6 in aluminum cages (Iffa-Credo, Saint-Germain sur l’Abresle, France) on straw and hay litter (Amazonie, Toulouse, France) with free access to food (Teklad 2040 global guinea-pig, batch M373; Harlan, Gannat, France) and water until tested. The animal house was maintained under artificial lighting (12 h) between 7:00 and 19:00 in a controlled ambient temperature of 21 ± 3°C, and relative humidity maintained in the range 40–70%.

### Guinea-Pig Isolated Urinary Bladder

Guinea-pigs were sacrificed by cervical dislocation, to avoid the possible effect of anesthetics on bladder smooth muscle response and/or drugs tested in experiments. The whole urinary bladder was excised and the detrusor muscle was dissected free from connective tissue and urothelium and cut into two equal strips isolated from the posterior face. Strips were mounted in 5 mL organ baths containing a Krebs–Henseleit solution and 0.3 μM GR159897, a selective NK_2_ receptor antagonist (Beresford et al., 1995), 1 μM propranolol and 1 μM indomethacin. A resting tension of 1 g was applied and contractile responses were measured using isometric tension transducers (EMKA Technologies, Paris, France) connected to amplifiers and to a data acquisition system (PowerLab 16s, ADInstruments, Sydney, NSW, Australia). Strips were first challenged with 80 mM KCl in order to test tissue viability. A cumulative concentration-response curve (CRC) to SP-OMe was performed between 1 nM and 10 μM. After wash-outs and 30 min of resting period, tissues were incubated with netupitant at 1, 3, 10, and 30 nM, or its solvent (0.03% ethanol) for 90 min, then a second CRC to SP-OMe was performed. In another series of experiments, a cumulative CRC to carbachol (0.1–100 μM) was performed, then tissues were incubated for 90 min with netupitant (300 nM) or the corresponding solvent before a second CRC to carbachol was constructed. Results were expressed as % of the maximal response obtained in the first CRC to the agonist. EC_50_ values for SP-OMe and carbachol in the absence and presence of each concentration of netupitant or solvent, were calculated by a non-linear regression and compared using the software Graph Pad Prism^®^ v 4.0 (GraphPad Softwares, La Jolla, CA, USA). Comparison between controls and treated CRC were performed. EC_50_ Dose-Ratios (DR) for SP-OMe or carbachol in the presence and absence of each concentration of netupitant or solvent were calculated. The potency of netupitant on NK_1_ receptors was calculated using the formula:

pKB = log(antagonist) + (doseratio - 1)

### Cystometric Studies

Guinea-pigs were anesthetized with urethane 1.5 g/kg (i.p.), a tracheotomy was performed and a PE cannula was inserted into the trachea. A polyethylene catheter (0.3 and 0.7 mm of internal and outer diameters, respectively) was implanted into the bladder through the dome. A catheter (0.58 and 0.96 mm of internal and outer diameters, respectively) was also introduced into a jugular vein for test substance administration. For isovolumetric recordings, the ureters were tied distally and cut to prevent the accumulation of urine in the bladder. Bladder catheters were connected via a T-tube to a strain gage (MX 860 Novatrans III Gold; Medex Médical Nantes-Carquefou, France) and to a single-syringe pump (70-2208 Model II Plus, Harvard Apparatus; Les Ulis, France). Vesical pressure was recorded continuously using a PowerLab interface (AD instruments) and the software Chart. Data were analyzed with Chart and Microsoft Excel softwares. At the end of the cystometric experiments, animals were killed by a lethal dose (100 mg/kg, i.p.) of pentobarbital followed by cervical dislocation.

#### Isovolumetric Model in Anesthetized Guinea-Pigs

The experimental protocol to evaluate the effects of test substances on lower urinary tract function in anesthetized guinea-pigs was adapted from a previous study (Doi et al., 1999). After anesthesia induction, the urethral meatus was ligated. Then, the bladder was filled with physiological saline by single injections of 0.5 mL every 5 min until rhythmic bladder contractions (RBC) occurred. After the occurrence of first two bladder contractions (to confirm the initiation of the RBC), a 30 min control period was allowed, prior to collecting basal values. Then, netupitant (0.1, 0.3, 1, or 3 mg/kg) or L-733,060 (3 or 10 mg/kg) or the corresponding vehicle were administered by i.v. infusion (1 mL at 0.2 mL/min). Vesical pressure was recorded for up to 90 min after administration. The parameters measured were bladder contraction frequency (BCF, number of RBC per interval of 30 min) and bladder contraction amplitude (BCA) calculated as maximal bladder pressure at RBC minus the intravesical pressure just before RBC; mmHg). For each guinea-pig, values for each parameter were calculated as mean of all bladder contractions observed during the first 30 min of cystometry (basal values) and following test substance administrations (three periods of 30 min: 0/30, 30/60, and 60/90 min). The effects of netupitant and L-733,060 were evaluated only for the first 30 min post-administration. All the results were expressed as % of basal values.

#### Acetic Acid Model in Anesthetized Guinea-Pigs

This experimental model was previously described by our laboratory (Kalinichev et al., 2014). Briefly, cystometric investigations were performed starting 15 min after surgery. The bladder catheter was connected via a T-tube to a strain gage and to a single-syringe injection pump. Physiological saline or 0.2 % AA were infused into the bladder at a constant flow rate (12 mL/hr). After a first micturition, cystometry was performed for 30 min in order to calculate basal values, then netupitant in doses between 0.1 and 3 mg/kg or L-733,060 (3 and 10 mg/kg) or the corresponding vehicle, were administered by i.v. infusion (1 mL; 0.2 mL/min). Vesical pressure was recorded for 1 h after administration. The cystometry parameters measured were: threshold pressure (ThP, pressure at which micturition occurs, mmHg), amplitude of micturition contractions (AM, pressure between ThP and micturition peak pressure, mmHg), intercontraction interval (ICI; time between two consecutive micturitions, min), micturition frequency (MF, number of micturition contractions per interval of 15 min, peaks/15 min) and basal pressure (BP, the lowest pressure immediately after micturition contraction, mmHg).

For each guinea-pig, basal values for each parameter were calculated as mean of 2–4 micturitions observed during the period of 30 min before test substance or vehicle administration. To construct dose-response relationship, the effects of netupitant and L-733,060 were evaluated for the first 15 min of post-administration. All the results were expressed as % of basal values.

#### Statistical Analysis

All values are means ± SEM Welch’s *t*-test, and Sidak’s multiple comparisons test following repeated measures two-way analysis of variance (ANOVA) were used for statistical analyses, if applicable. For all analyses, *P* < 0.05 was considered statistically significant.

#### Chemicals

Acetic acid, absolute ethanol, carbamoylcholine chloride (carbachol), (±)-propranolol hydrochloride, indomethacin and urethane were obtained from Sigma-Aldrich (L’Isle d’Abeau Chesnes, Saint Quentin Fallavier, France). GR 159897, Substance P methyl ester (SP-OMe) and L-733,060 hydrochloride (MW = 439.83) were obtained from Tocris Bioscience (Moorend Farm Avenue, Bristol, U.K.). Physiological saline (NaCl 0.9%) and pentobarbital were purchased from Centravet (Lapalisse, France). Glucose, propylene glycol and HCl 2N were purchased from VWR International (Le Perigares, Fontenay-sous-Bois, France). Stock solutions of SP-OMe at the concentration of 10 mM were prepared in distilled water. Aliquots of SP-OMe were stored at -20°C. Fresh solutions of carbachol at 10 mM were prepared on the day of experimentation. Dilutions were prepared in distilled water. Netupitant (MW = 578.61) was synthesized by the Medicinal Chemistry Department of Helsinn Healthcare. For *in vitro* studies, netupitant was dissolved in pure ethanol, and then diluted in distilled water.

For *in vivo* studies, a stock solution of netupitant at the concentration of 50 mg/mL was prepared by dissolving 100 mg of netupitant in 2N HCl, followed by adding pure propylene glycol to make 2 mL in total volume. This solution was stored at 20°C (protected from light). On the day of experimentation the stock solution of netupitant was diluted in 5% glucose according to each animal’s body weight so that each animal would receive each drug dose in an equal volume (1 mL). The pH was 6.0. Likewise, L-733,060 was dissolved in 5% glucose.

## Results

### Guinea-Pig Isolated Urinary Bladder

**FIGURE 1:**
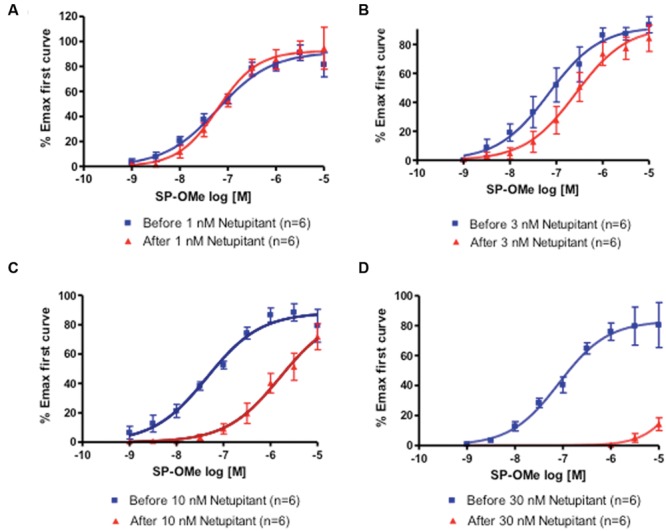
**First and second concentration-response curves (CRCs) to SP-OMe in guinea-pig isolated urinary bladders incubated with netupitant at **(A)** 1 nM; **(B)** 3 nM; **(C)** 10 nM; **(D)** 30 nM**.

Two consecutive CRCs to SP-OMe (*n* = 6) in the same detrusor muscle strip were reproducible in terms of agonist potency. In fact, pEC_50_ values for SP-OMe were 7.73 ± 0.12 (first CRC) and 7.56 ± 0.10 (second CRC following solvent incubation), which shows no difference between the two values. Netupitant inhibited SP-OMe induced contractions in a concentration-dependent manner (**Figures 1A–D**). Netupitant at 1 nM did not affect the second CRC to SP-OMe, however, 3 and 10 nM produced parallel rightward shifts in the CRCs to SP-OMe without affecting the maximal response (Emax). At 30 nM, netupitant totally depressed the second CRC (**Figure 1D**) making it impossible to use a Schild plot to calculate the antagonist potency value. The pK_B_ value for netupitant was estimated to be 8.95–9.54 (mean 9.24) using displacement obtained at 3 and 10 nM, respectively.

Two consecutive CRCs to carbachol (*n* = 6) in the same detrusor muscle strip were reproducible and not affected by solvent incubation. In fact, pEC_50_ values for carbachol, 6.21 ± 0.05 (first CRC) and 6.10 ± 0.05 (second CRC) were not statistically different, as well as Emax values (data not shown). Netupitant at 300 nM had no effect on the CRC to carbachol (data not shown).

### Isovolumetric Model in Anesthetized Guinea-Pigs

**FIGURE 2:**
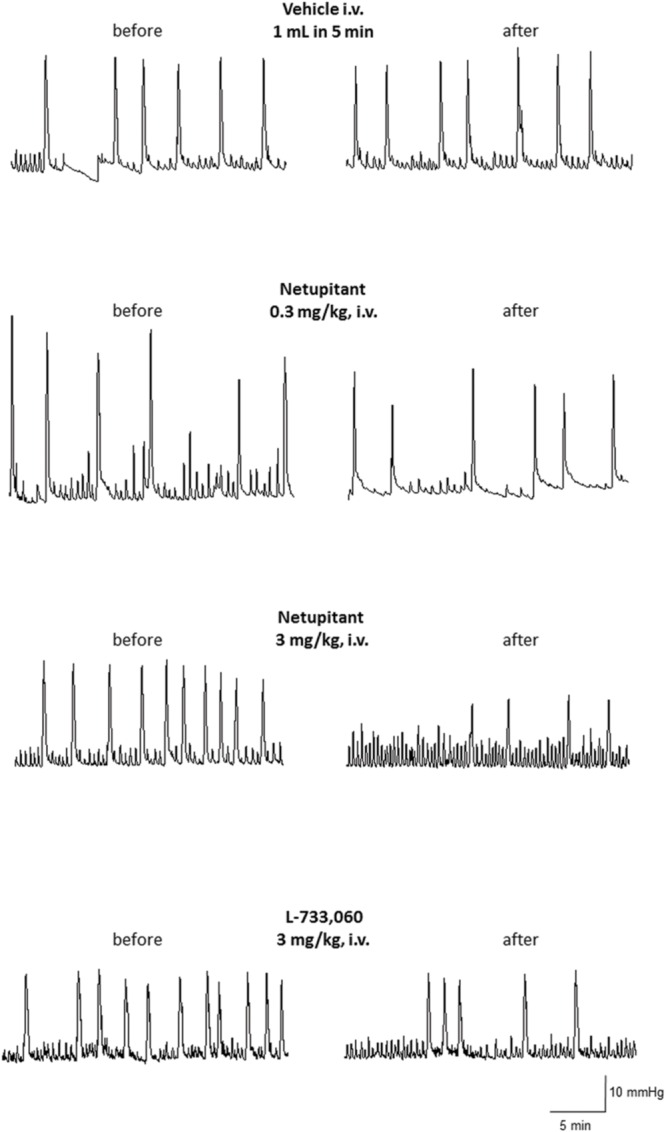
**Cystometric recordings in the isovolumetric model obtained following administration of vehicle, netupitant at 0.3 and 3 mg/Kg, i.v. and L-733,060 at 3 mg/kg, i.v**.

Repeated cystometries showed reproducible results in all experimental groups. No differences were found in basal values of BCF and BCA among the groups that received different drug dose or vehicle (data not shown). **Figure 2** shows typical cystometric recordings obtained following administration of vehicle, netupitant at 0.3 and 3 mg/kg i.v. and L-733,060 at 3 mg/kg, i.v.

**FIGURE 3:**
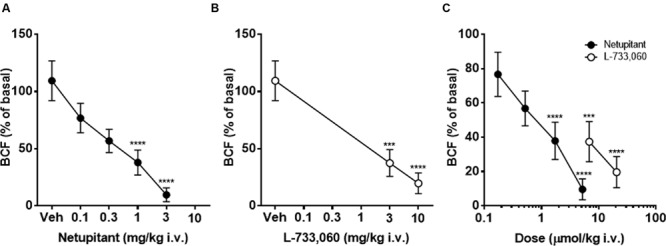
**Cystometry under isovolumetric conditions in female guinea-pigs**. Graph showing the effects of graded doses of netupitant **(A)** (*n* = 10 for each dose) and L-733,060 **(B)** (*n* = 10 for each dose) on bladder contraction frequency (BCF). Doses of netupitant and L-733,060 are expressed in μmol/kg to compare the potencies of these drugs on BCF **(C)**. Asterisks indicate statistical difference from the basal (^∗∗∗^*P* < 0.001, ^∗∗∗∗^*P* < 0.0001, Sidak’s multiple comparison test following repeated measures two-way ANOVA). All statistical analyses were performed using actual values.

Intravenous administration of vehicle did not significantly change BCF for 90 min after the administration (data not shown). Because in the majority of animals that received netupitant or L-733,060, the maximal effects appeared within 30 min of the injection, the drug effects were compared at 30 min post-administration. Netupitant dose-dependently reduced BCF in a statistically significant manner, both at 1 and 3 mg/kg (**Figure 3A**). In the period 0–30 min post-administration L-733,060 dose-dependently reduced BCF in a statistically significant manner, both at 3 and 10 mg/kg (**Figure 3B**). In **Figure 3C**, doses are expressed in μmol/kg in order to precisely compare netupitant and L-733,060 potencies for the effects on BCF. From these data, we can estimate that netupitant is approximately 3.9 times as potent as L-733,060.

**FIGURE 4:**
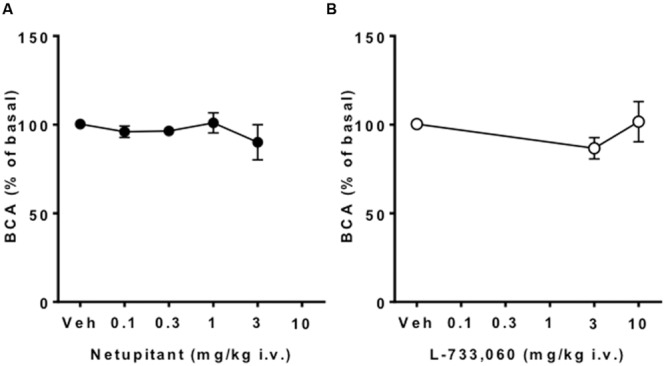
**Effect of netupitant or L-733,060 on BCA (*n* = 10 for each).** Neither drug at any doses changed BCA.

Meanwhile, neither netupitant nor L-733,060 significantly changed BCA because both drugs produced variable effects on this parameter (**Figures 2** and **4**).

### AA Model in Anesthetized Guinea-Pigs

In comparison to saline infusion, 0.2% AA significantly increased AM and MF, and decreased ICI (**Table 1**). ThP and BP were not affected by 0.2% AA bladder infusion (**Table 1**).

**Table 1:** Comparisons in cystometry variables between saline infusion and acetic acid infusion.

Basal	ThP (mmHg)	AM (mmHg)	BP (mmHg)	ICI (s)	BC (ml)	AUC (mmHg.s)	MF (peaks/15 min)
Saline (*n* = 10)	14.0 ± 1.0	6.2 ± 0.3	10.5 ± 1.0	671 ± 68	2.23 ± 0.23	2329 ± 269	1.4 ± 0.2
AA (*n* = 70)	12.1 ± 0.3	16.3 ± 0.6^∗∗∗^	11.7 ± 0.3	200 ± 9^∗∗∗^	0.67 ± 0.03^∗∗∗^	3065 ± 149^∗^	4.7 ± 0.2^∗∗∗^

*ThP, threshold pressure for inducing micturition contraction; AM, amplitude of micturition contraction; BP, basal pressure; ICI, intercontraction interval; BC, bladder capacity; AUC, area under curve; MF, micturition frequency. ^∗^*P* < 0.05, ^∗∗∗^*P* < 0.001 (Welch’s *t*–test).*

**FIGURE 5:**
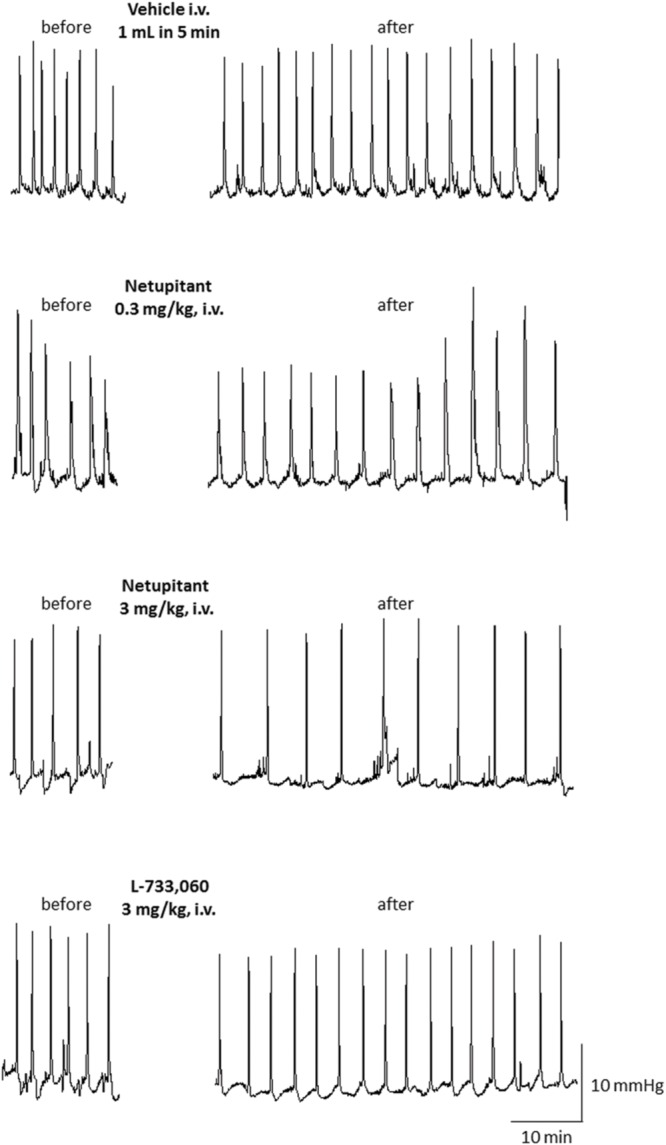
**Cystometric recordings in the acetic acid model obtained following administration of vehicle, netupitant at 0.3 and 3 mg/kg i.v. and L-733,060 at 3 mg/kg, i.v**.

**FIGURE 6:**
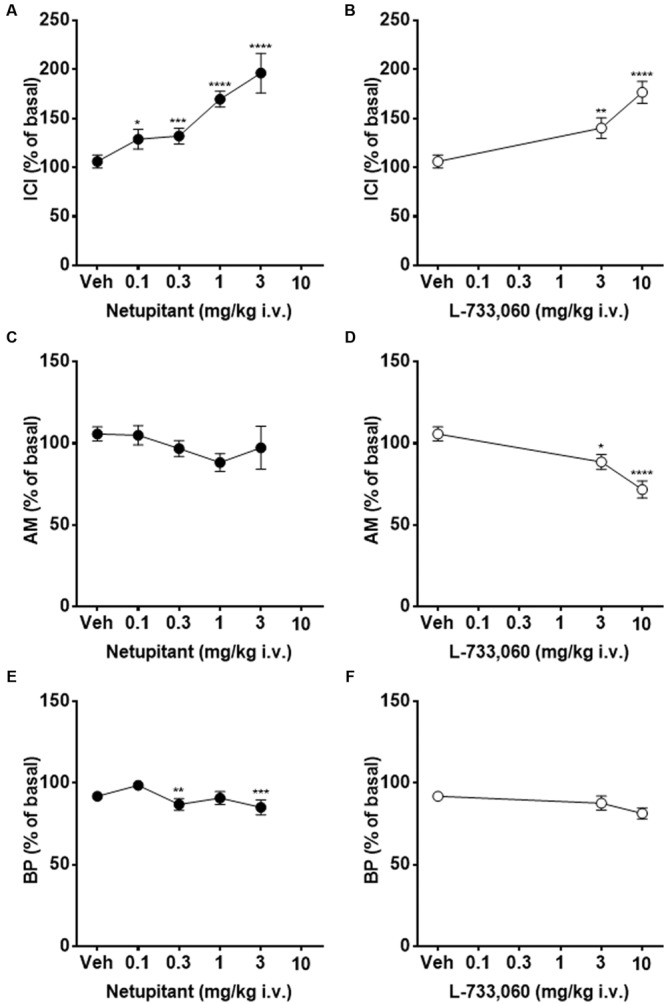
**Continuous acetic acid infusion CMGs in female guinea-pigs**. Graphs showing the effects of graded doses of netupitant on ICI **(A)**, AM **(C)**, and BP **(E)** (*n* = 10 for each), and those of L-733,060 on ICI **(B)**, AM **(D)**, and BP **(F)** (*n* = 10 for each). Asterisks indicate statistical difference from the basal (^∗^*P* < 0.05, ^∗∗^*P* < 0.01, ^∗∗∗^*P* < 0.001, ^∗∗∗∗^*P* < 0.0001, Sidak’s multiple comparison test following repeated measures two-way ANOVA). All statistical analyses were performed using actual values.

**Figure 5** shows typical cystometric recordings obtained following administration of vehicle, netupitant at 0.3 and 3 mg/kg, i.v. and L-733,060 at 3 mg/kg, i.v. Netupitant in the range 0.1–3 mg/kg i.v., significantly increased ICI (within 15 post-administration) and these effects were statistically significant at all doses examined (**Figure 6A**). Netupitant at any doses did not change AM (**Figure 6C**) and at 0.3 and 3 mg/kg decreased BP (**Figure 6E**). ThP was not modified by any dose of netupitant with respect to vehicle administration (data not shown).

L-733,060 at 3 and 10 mg/kg i.v., significantly increased ICI (within 15 post-administration) (**Figure 6B**). Moreover, AM (**Figure 6D**) was significantly decreased by L-733,060 at 3 and 10 mg/kg, whereas BP (**Figure 6F**) and ThP (data not shown) were not changed.

**FIGURE 7:**
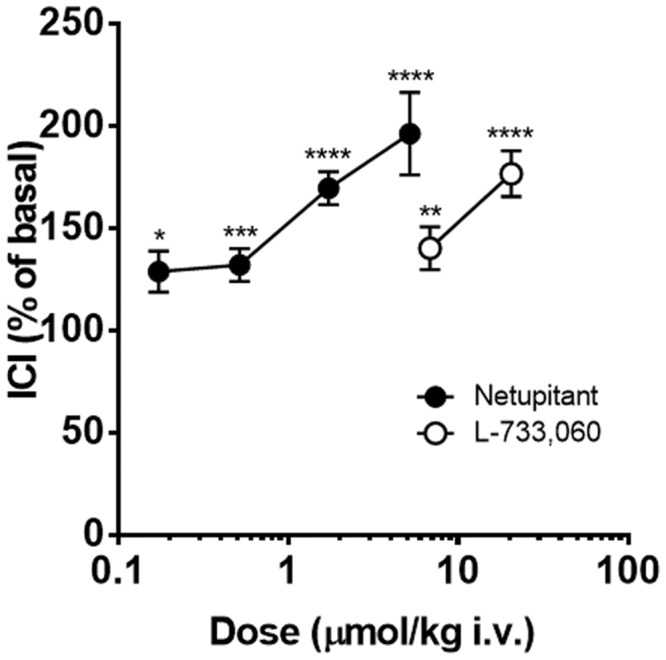
**Continuous acetic acid infusion CMGs in female guinea-pigs.** Graphs showing the effects of netupitant (*n* = 10 for each dose) or L-733,060 (*n* = 10 for each dose) on ICI. Doses of these drugs are expressed in μmol/kg to compare the potencies of these drugs on ICI. Asterisks indicate statistical difference from the basal (^∗^*P* < 0.05, ^∗∗^*P* < 0.01, ^∗∗∗^*P* < 0.001, ^∗∗∗∗^*P* < 0.0001, Sidak’s multiple comparison test following repeated measures two-way ANOVA). All statistical analyses were performed using actual values.

Finally, a direct comparison of the potency of netupitant and L-733,060 on ICI, when doses were expressed as μmol/kg, is reported in **Figure 7**, showing that netupitant is approximately 11 times as potent as L-733,060.

## Discussion

### *In vitro* Results

Our results demonstrate that netupitant is a potent antagonist of the detrusor muscle contractions evoked by the activation of NK_1_ receptors in the guinea-pig isolated urinary bladder. The potency of netupitant (mean pK_B_ = 9.24) was very similar to its reported affinity for human recombinant NK_1_ receptors (pK_i_ = 9.01; Hoffmann et al., 2006) confirming that the pharmacology of the guinea-pig NK_1_ receptors is very similar to human receptors, as previously suggested (Saria, 1999). At low concentrations netupitant showed a competitive antagonism based on the parallel rightward shifts in the CRCs to SP-OMe observed. However, at concentrations greater than 10 nM, netupitant strongly reduced Emax values. It was not possible, for practical reasons, to increase the concentrations of the agonist up to 10 μM (because of the limited solubility of SP-OMe into the Krebs solution) so we cannot conclude, from the present experiments, the nature of the antagonism. It should be noted, however, that in previous investigations on CHO NK_1_ cells, netupitant concentration-dependently antagonized the stimulatory effects of SP showing insurmountable antagonism (pK_B_ = 8.87; Rizzi et al., 2012).

Netupitant at 300 nM had no effect on the contractile response to carbachol, confirming its selectivity for NK_1_ receptors.

#### Isovolumetric Model

The aim of this study was to compare the effects of two NK_1_ receptor antagonists on cystometric parameters in a model of urinary bladder function (under isovolumetric conditions) in anesthetized female guinea-pigs. In agreement with the literature, isovolumetric conditions elicited RBCs, stable in frequency and amplitude (Lecci et al., 1993; Yoshiyama and de Groat, 2001; Nagabukuro et al., 2004). After vehicle administration, BCF and BCA were not changed. Therefore, in this set of experiments, we can exclude confounding factors interfering with the evaluation of the effects of netupitant and L-733,060. Although in some animals BCA values following netupitant at the highest dose tested (3 mg/kg) were quite variable (see **Figures 2** and **4**), overall we did not observe any statistically significant change in BCA following netupitant administration. Therefore, we conclude that an *in vivo* anti-muscarinic property or a direct inhibitory action on bladder smooth muscle of netupitant can be excluded at doses used in this study. The doses of netupitant were chosen based on previous *in vivo* experiments in different animal species (Hoffmann et al., 2006; Rizzi et al., 2012). The doses of L-733,060 were chosen based on a previous paper showing that, in guinea-pigs, this antagonist at 10 mg/kg i.p. increased latency to immobility in the forced swimming test (Wicke et al., 2007). Netupitant, dose-dependently reduced BCF after i.v. administration and the inhibition was statistically significant at 1 and 3 mg/kg. These results are in agreement with previous results obtained in guinea-pigs with another NK_1_ antagonist (TAK-637) since its systemic administration decreased the number but not the amplitude of the distension-induced RBC (Doi et al., 2000). The same group, following several experiments, concluded that TAK-637 inhibits the micturition reflex by acting, at least in part, on the spinal cord (Kamo et al., 2000). The site of action of netupitant was not investigated in the present experiments, however, in analogy with TAK-637, and considering the high penetration of this molecule into the CNS, we advance the hypothesis that netupitant-induced inhibition of RBC could be, at least in part, mediated by structures inside the spinal cord and/or the brain. It is interesting to note that first dose of netupitant having a significant effect on BCF (1 mg/kg, i.v.) in the isovolumetric model is quite close to the value obtained in a behavioral model in gerbils (foot tapping behavior evoked by the intracerebroventricular injection of a NK_1_ agonist). In this model netupitant displayed an ID_50_ value of 1.5 mg/kg, i.p. (Rizzi et al., 2012).

As a comparison of drug potency, using the same experimental model in anesthetized guinea-pigs, we have shown that the novel GABA_B_ receptor positive allosteric modulator ADX71441, significantly reduced BCF in the dose range 1–3mg/kg, i.v, so similar to the netupitant doses (Kalinichev et al., 2014). It should be noted, however, that at 3mg/kg ADX71441 completely inhibited the micturition reflex and induced overflow incontinence in 50% of guinea-pigs, an effect potentially negative for the management of OAB patients.

Netupitant (3 mg/kg i.v.) produced a greater decrease in BCF compared to the effect of L-733,060 at the same dose. A direct comparison of the effects of the two compounds, when doses were expressed as μmol/kg, showed that netupitant is approximately 4 times more potent than L-733,060. This last molecule, on a functional test in recombinant hNK_1_ receptors, inhibited SP-induced calcium mobilization with an estimated affinity of 0.8 nM (Seabrook et al., 1996) a value close to the netupitant potency *in vitro* (pK_B_ = 9.24 in guinea-pig detrusor muscle). Therefore, since netupitant and L-733,060 has approximately the same potency *in vitro*, the greater potency of netupitant *in vivo* could be explained by greater bioavailability or a better penetration of netupitant into guinea-pig CNS. This last hypothesis is supported by the high level of brain penetration and receptor occupancy (90%) in human volunteers following different doses of netupitant (Spinelli et al., 2014).

#### Acetic Acid Results

In cats and rats, it is well known that intravesical infusion of diluted AA elicits bladder overactivity characterized by an increase in MF (Yoshiyama et al., 1993; Thor et al., 2002). Our results show that intravesical infusion of diluted AA (0.2%) elicits a significant increase in MF in guinea-pigs also, associated with a significant increase in AM. Again, no effect of vehicle was observed in guinea-pigs with 0.2% AA bladder infusion. Therefore, in this set of experiments, we can exclude confounding factors interfering with the evaluation of the effects of netupitant and L-733,060 in this experimental model.

In guinea-pigs with AA bladder infusion, the two NK_1_ receptor antagonists increased ICI at 15 min post-administration in a dose-dependent manner. Netupitant administration reached statistical significance starting from 0.3 mg/kg, i.v. whereas L-733,060 had a significant effect at 3 and 10 mg/kg, suggesting that netupitant is approximately 10 times more potent than L-733,060.

In rats with intravesical infusion of diluted AA, capsaicin desensitization prevents bladder overactivity, suggesting the activation of capsaicin-sensitive afferent fibers by AA (Mitsui et al., 2001). Since SP is present in capsaicin-sensitive afferent fibers (Andersson, 1999), the decrease in bladder overactivity observed in this study could be explained, at least in part, by a peripheral action of netupitant to inhibit bladder afferent activity or by a central action to suppress transmission at afferent synapses in the spinal cord, or both. SP is known to increase the excitability of bladder afferents in rats (Chien et al., 2003). Interestingly, a selective NK_1_ receptor agonist increased the excitability of L6-S1 dorsal root ganglia isolated from female guinea-pigs and this effect was blocked by 200 nM netupitant (Zhang et al., 2012).

Amplitude of micturition was not affected by any dose of netupitant tested. The lack of effect of netupitant on AM is in agreement with *in vitro* studies, showing that netupitant at a concentration 300 times greater than its pK_i_ value was ineffective as an antagonist of carbachol-induced detrusor muscle contractions. However, L-733,060, at 3 and 10 mg/kg i.v., significantly reduced AM. This result suggests that L-733,060 can interfere with the contractile machinery of the urinary bladder, presumably by an antimuscarinic effect or an effect on calcium channels. The reduction of AM should be avoided in OAB patients, since this could generate a condition called bladder underactivity and inducing difficulties to void. Therefore, the experimental model here described can allow the selection of drug candidates showing a significant increase on ICI but without the inhibitory effect on AM.

Several laboratories has advanced the hypothesis that NK_1_ receptor antagonists, by blocking neurotransmission through afferent fibers could be the drug of choice for treating overactive bladder (Yoshimura et al., 2008). The potent inhibitory effect of NK_1_ receptor antagonists on BCF in anesthetized guinea-pigs suggests that NK_1_ receptors are involved in the afferent limb of the micturition reflex. Because of its novel mechanism of action coupled with its selectivity over muscarinic receptors, netupitant could produce positive effects on bladder function in overactive bladder patients without reductions in bladder contractility and salivary secretion. These side effects are frequently observed with antimuscarinics, a class of drugs considered the gold standard for the treatment of urinary incontinence and OAB. Recently, netupitant was tested on a phase II, multicenter, double-blind study. Males and females patients with OAB symptoms >6 months were randomized to receive one of 3 doses of netupitant (50, 100, 200 mg) or placebo once daily for 8 weeks. Unfortunately, the study failed to demonstrate superiority of netupitant *versus* placebo in decreasing OAB symptoms, despite a trend favoring netupitant 100 mg (Haab et al., 2014). This failure evidences the difficulties to translate basic research from animals to human pathologies, and the need to develop more pertinent experimental models for OAB. However, it should be noted that the clinical study was performed on patients with high heterogeneity with respect to sex, overall symptoms and the main urinary tract dysfunction, suggesting that future studies on OAB should carefully consider the stratifications of the patients according to more homogeneous characteristics.

It is well known that NK_1_ receptors are involved in cellular responses such as pain transmission as well as endocrine and paracrine secretion (Garcia-Recio and Gascón, 2015). SP-containing nerve fibers in human bladder were increased, with respect to controls, in the submucosa of interstitial cystitis (IC) patients and were frequently observed in juxtaposition to mast-cells (Pang et al., 1995). Therefore, netupitant could have a positive effect on chronic inflammation and pelvic pain in IC patients. However, this hypothesis should be tested in randomized clinical trials.

## Author Contributions

VG, MR, MG, and M-AG contributed in generating experimental data. SP, VG, PL, and CP contributed in designing the experimental protocols. MR, VG, and MY analyzed raw data and performed statistical analysis. SP, EL, CP, and MY contributed in discussion and reviewed the manuscript. SP and MY wrote the manuscript and drew the figures.

## Conflict of Interest Statement

The authors declare that the research was conducted in the absence of any commercial or financial relationships that could be construed as a potential conflict of interest.
